# Supplementary material for: Direct generation of entangled photon pairs in nonlinear optical waveguides
Source: Nanophotonics. 2022 Jan 21;11(5):1021–32. doi: 10.1515/nanoph-2021-0736 (PMC11501258; doi:10.1515/nanoph-2021-0736)
Supplement: Supplementary file 1 — Supplementary Material Details [file j_nanoph-2021-0736_suppl.pdf]

Direct generation of entangled photon pairs in nonlinear optical waveguides
– SUPPLEMENTARY INFORMATION –

Álvaro Rodríguez Echarri,[1] Joel D. Cox,[2,3] and F. Javier García de Abajo[1,4,*]

[1]*ICFO-Institut de Ciencies Fotoniques, The Barcelona Institute of Science and Technology, 08860 Castelldefels (Barcelona), Spain*
[2]*Center for Nano Optics, University of Southern Denmark, Campusvej 55, DK-5230 Odense M, Denmark*
[3]*Danish Institute for Advanced Study, University of Southern Denmark, Campusvej 55, DK-5230 Odense M, Denmark*
[4]*ICREA-Institució Catalana de Recerca i Estudis Avançats, Passeig Lluís Companys 23, 08010 Barcelona, Spain*

We plot the mode dispersion relations and group velocities in a freestanding waveguide of permittivity $\epsilon_1 = 1$ and present details of the theory used in the main text. In particular, we discuss the reflection and transmission coefficients for outgoing waves emanating from the waveguide material, the decomposition of a light plane wave and a line-dipole field in cylindrical waves, and the far-field limit of the electromagnetic Green tensor. Additionally, the modes of cylindrical, square, and rectangular waveguides are compared.

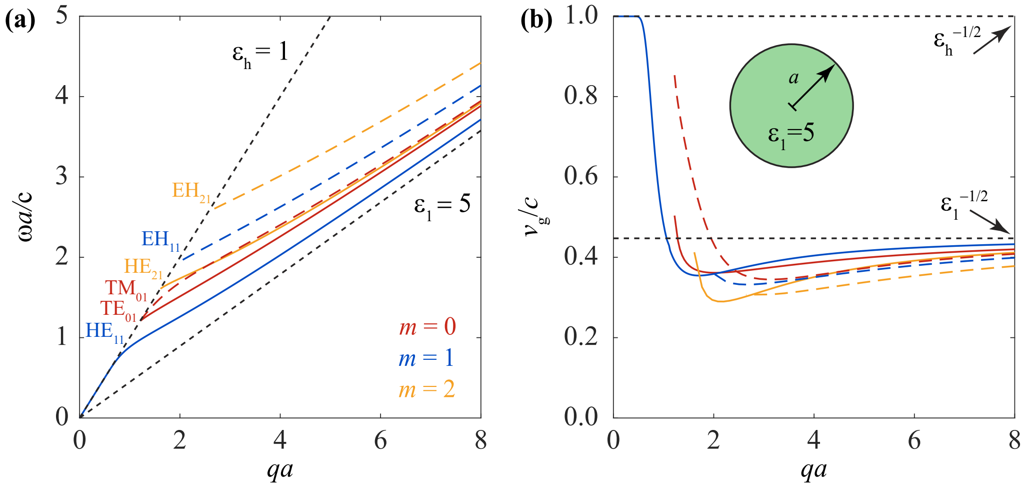

FIG. S1: **Dispersion relation of guided modes.** **(a)** Dispersion relations of the lowest-orders propagating modes in a cylindrical waveguide of radius a made of $\epsilon_1 = 5$ material and hosted in air ($\epsilon_h = 1$). We consider the lowest-order solutions with azimuthal and radial numbers $m = 0 - 2$ and $l = 1$. Mode labels follow the notation TE_{0l} and TM_{0l} for $m = 0$ (transverse electric and magnetic modes, respectively), as well as EH_{ml} and HE_{ml} for $m \neq 0$ (see Sec. 4.1.1 in the main text). **(b)** Group velocities corresponding to the modes in (a). Black dashed lines in both panels indicate the light cones inside and outside the waveguide.

S1. REFLECTION AND TRANSMISSION COEFFICIENTS OF INNER CYLINDRICAL WAVES AT THE WAVEGUIDE INTERFACE

Following the notation introduced in Sec. 4.1 of the main text, we first note that m and q are unchanged upon reflection or transmission due to the cylindrical symmetry of the waveguide. The corresponding coefficients $r_{m,\sigma'\sigma}$ and $t_{m,\sigma'\sigma}$ for a cylindrical wave of electric field $\mathbf{E}^H_{1,qm\sigma}$ and polarization $\sigma \in \{s,p\}$ emanating from inside the waveguide are defined through the expression

$$\mathbf{E} = \begin{cases} \mathbf{E}^H_{1,qm\sigma} + r_{m,s\sigma}\mathbf{E}^J_{1,qms} + r_{m,p\sigma}\mathbf{E}^J_{1,qmp}, & R < a, \\ t_{m,s\sigma}\mathbf{E}^H_{h,qms} + t_{m,p\sigma}\mathbf{E}^H_{h,qmp}, & R \geq a, \end{cases}$$

*Electronic address: javier.garciadeabajo@nanophotonics.es

where the reflected waves $\mathbf{E}^J_{1,qm\sigma'}$ are regular propagating solutions inside the waveguide material $j=1$, while the transmitted fields $\mathbf{E}^H_{h,qm\sigma'}$ are outgoing solutions in the host medium $j=h$. We now enforce the continuity of the tangential components (i.e., perpendicular to $\hat{\mathbf{R}}$) in both magnetic and electric fields, where the former is obtained from the latter by using Faraday's law ($\mathbf{H}^{J/H}_{j,qm\sigma}=(1/\mathrm{i}k)\nabla\times\mathbf{E}^{J/H}_{j,qm\sigma}$) combined with the identity [1] $k_j\mathbf{E}^{J/H}_{j,qm\sigma}=\nabla\times\mathbf{E}^{J/H}_{j,qm\sigma'}$, valid for $\sigma\neq\sigma'$. This leads to the expressions

$$\left[r_{m,s\sigma}\mathbf{E}^J_{1,qms}-t_{m,s\sigma}\mathbf{E}^H_{h,qms}+r_{m,p\sigma}\mathbf{E}^J_{1,qmp}-t_{m,p\sigma}\mathbf{E}^H_{h,qmp}\right]\times\hat{\mathbf{R}}=\;-\mathbf{E}^H_{1,qm\sigma}\times\hat{\mathbf{R}}, \tag{S1a}$$

$$\left[\zeta r_{m,s\sigma}\mathbf{E}^J_{1,qmp}-t_{m,s\sigma}\mathbf{E}^H_{h,qmp}+\zeta r_{m,p\sigma}\mathbf{E}^J_{1,qms}-t_{m,p\sigma}\mathbf{E}^H_{h,qms}\right]\times\hat{\mathbf{R}}=-\zeta\mathbf{E}^H_{1,qm\sigma'}\times\hat{\mathbf{R}}, \tag{S1b}$$

where $\zeta=\sqrt{\epsilon_1/\epsilon_h}$ and $\sigma\neq\sigma'$. By inserting the explicit expressions of the mode fields into Eqs. (S1) and projecting on $\hat{\varphi}$ and $\hat{\mathbf{z}}$ components, we readily find the secular matrix M and the linear equations for the reflection and transmission coefficients given in Sec. 4.1. Waveguide modes are signalled by the zeros of det$\{M\}$, as discussed in Sec. 4.1.1 of the main text. We show the dispersion relation and group velocity of the lowest-order modes for $\epsilon_1=5$ and $\epsilon_\mathrm{h}=1$ in Figure S1.

S2. DECOMPOSITION OF A LIGHT PLANE WAVE IN CYLINDRICAL WAVES

We work in frequency space ω and consider a light plane wave propagating in a medium of permittivity ϵ_1 with unit electric field $\hat{\mathbf{e}}^\pm_\sigma\mathrm{e}^{\mathrm{i}\mathbf{k}^\pm_1\cdot\mathbf{r}}$ of polarization $\sigma\in\{s,p\}$ and wave vector $\mathbf{k}^\pm_1=\mathbf{Q}\pm k_{1z}\hat{\mathbf{z}}$. Here, $\mathbf{Q}=(Q_x,Q_y)=(Q,\varphi_\mathbf{Q})$, $k_{1z}=\sqrt{\epsilon_1k^2-Q^2+\mathrm{i}0^+}$ (with $k=\omega/c$ and $\mathrm{Re}\{k_{1z}\}>0$), and the polarization vectors are defined as $\hat{\mathbf{e}}^\pm_s=(-Q_y\hat{\mathbf{x}}+Q_x\hat{\mathbf{y}})/Q$ and $\hat{\mathbf{e}}^\pm_p=(\pm\mathbf{Q}k_{1z}-Q^2\hat{\mathbf{z}})/k_1Q$. It is convenient to recast the dependence on $\mathbf{R}=(x,y)=(R,\varphi)$ by using the orthogonality relation $\int_0^\infty RdR\,J_m(QR)J_m(Q'R)=\delta(Q-Q')/Q$ together with the integral $\int_0^{2\pi}d\varphi\,\mathrm{e}^{\mathrm{i}\mathbf{Q}\cdot\mathbf{R}}\mathrm{e}^{\mathrm{i}m\varphi}=2\pi\mathrm{i}^mJ_m(QR)\mathrm{e}^{\mathrm{i}m\varphi_\mathbf{Q}}$, from which we derive the Fourier expansion $\mathrm{e}^{\mathrm{i}\mathbf{Q}\cdot\mathbf{R}}=\sum_m\mathrm{i}^mJ_m(QR)\,\mathrm{e}^{\mathrm{i}m(\varphi-\varphi_\mathbf{Q})}$. Combining this result together with the explicit forms of the polarization vectors given above, we can assimilate each of the m terms in the Fourier transform of the plane wave field to a cylindrical wave and write

$$\hat{\mathbf{e}}^\pm_s\mathrm{e}^{\mathrm{i}\mathbf{k}^\pm_1\cdot\mathbf{r}}=\sum_m\mathrm{i}^{m+1}\,\mathrm{e}^{-\mathrm{i}m\varphi_\mathbf{Q}}\,\mathbf{E}^J_{1,\pm k_{1z}ms},$$
$$\hat{\mathbf{e}}^\pm_p\mathrm{e}^{\mathrm{i}\mathbf{k}^\pm_1\cdot\mathbf{r}}=-\sum_m\mathrm{i}^{m}\,\mathrm{e}^{-\mathrm{i}m\varphi_\mathbf{Q}}\,\mathbf{E}^J_{1,\pm k_{1z}mp},$$

where $\mathbf{E}^J_{1,qm\sigma}$ is defined in Sec. 4.1 in the main text.

S3. DECOMPOSITION OF THE FIELD DUE TO A LINE DIPOLE IN CYLINDRICAL WAVES

In Sec. 4.2 of the main text, we express the field produced by a line dipole $\mathbf{p}$ in a homogeneous medium ϵ_1 as

$$\mathbf{E}^\mathrm{dip}(\mathbf{r},\mathbf{R}_0)=\frac{\mathrm{i}\pi}{\epsilon_1}\sum_mJ_m(Q_1R_0)\mathrm{e}^{-\mathrm{i}m\varphi_0}\left[k_1^2\mathbf{p}+\nabla(\mathbf{p}\cdot\nabla)\right]H^{(1)}_m(Q_1R)\,\mathrm{e}^{\mathrm{i}m\varphi}\mathrm{e}^{\mathrm{i}qz},$$

where the $\mathrm{e}^{\mathrm{i}qz}$ dependence is inherited from the modulation of the line dipole along z. We can express this field in terms of cylindrical waves by projecting the dipole on the circular coordinate vectors $\hat{\mathbf{e}}^\pm=(\hat{\mathbf{x}}\pm\mathrm{i}\hat{\mathbf{y}})/\sqrt{2}=\mathrm{e}^{\pm\mathrm{i}\varphi}(\hat{\mathbf{R}}\pm\mathrm{i}\hat{\varphi})/\sqrt{2}$, such that

$$\mathbf{p}=p_+\hat{\mathbf{e}}^++p_-\hat{\mathbf{e}}^-+p_z\hat{\mathbf{z}}$$

with coordinates

$$p_\pm=\mathbf{p}\cdot\hat{\mathbf{e}}^\mp=\mathbf{p}\cdot(\hat{\mathbf{x}}\mp\mathrm{i}\hat{\mathbf{y}})/\sqrt{2},$$
$$p_z=\mathbf{p}\cdot\hat{\mathbf{z}}.$$

Working out the p_z contribution to $\mathbf{E}^\mathrm{dip}$ and comparing it to the cylindrical waves in Sec. 4.1, we find that it reduces to $(\mathrm{i}\pi p_zQ_1k^2/k_1)\mathbf{E}^H_{1,qmp}$. The $p_\pm$ contributions are more involved, as they contain both p and s waves. More precisely, the $\mathbf{E}^\mathrm{dip}$ field that they generate has a $\hat{\mathbf{z}}$ component, which can be assigned to a p cylindrical wave $\propto\mathbf{E}^H_{1,q(m\pm1)p}$.

Adding and subtracting this wave to eliminate the z component, we find that the remaining $\hat{\mathbf{R}}$ and $\hat{\varphi}$ components reduce to a combination waves $\propto \mathbf{E}^H_{1,q(m\pm1)s}$ of s polarization. Combining these results, we can write the dipole field as

$$\mathbf{E}^{\rm dip}(\mathbf{R},\mathbf{R}_0)=\pi k^2\sum_m J_m(k_1R_0)\mathrm{e}^{-\mathrm{i}m\varphi_0}\left[\sum_\pm \frac{p_\pm}{\sqrt{2}}\left(\mathbf{E}^H_{1,q(m\pm1)s}\pm\frac{q}{k_1}\mathbf{E}^H_{1,q(m\pm1)p}\right)+\mathrm{i}p_z\frac{Q_1}{k_1}\mathbf{E}^H_{1,qmp}\right],$$

which is the expression reproduced in Sec. 4.2. In the algebraic manipulations needed to carry out these derivations, we make intensive use of the relations

$$\begin{aligned}\frac{m}{\theta}\mathcal{C}_m&=\frac{1}{2}(\mathcal{C}_{m-1}+\mathcal{C}_{m+1}),\\ \mathcal{C}'_m&=\frac{1}{2}(\mathcal{C}_{m-1}-\mathcal{C}_{m+1}),\\ \mathcal{C}''_m&=-\mathcal{C}_m+\frac{m-1}{2\theta}\mathcal{C}_{m-1}+\frac{m+1}{2\theta}\mathcal{C}_{m+1}\end{aligned}$$

for the Bessel and Hankel functions $\mathcal{C}_m(\theta)$, which can be directly obtained from the recurrence relation $\mathcal{C}'_m(\theta)=\pm(m/\theta)\mathcal{C}_m(\theta)\mp\mathcal{C}_{m\pm1}(\theta)$ [2].

S4. FAR-FIELD LIMIT OF THE ELECTROMAGNETIC GREEN TENSOR

Because the waveguide is translationally invariant along z, the Green tensor satisfies the identity

$$\mathcal{G}(\mathbf{r},\mathbf{r}',\omega)=\mathcal{G}(\mathbf{r}-z'\hat{\mathbf{z}},\mathbf{R}',\omega)\xrightarrow[k_{\rm h}r\gg1]{}\frac{\mathrm{e}^{\mathrm{i}k_{\rm h}r}}{r}\mathrm{e}^{-\mathrm{i}k_{\rm h}zz'/r}\mathbf{g}(\hat{\mathbf{r}},\mathbf{R}',\omega),\tag{S2}$$

where the rightmost expression represents the far-field limit in the host medium, for which we implicitly define a tensor $\mathbf{g}(\hat{\mathbf{r}},\mathbf{R}',\omega)$ that depends only on the direction of $\mathbf{r}$. In the derivation of this result, we have approximated $|\mathbf{r}-z'\hat{\mathbf{z}}|\approx r-zz'/r$ in the leading exponential, assuming that we have $r\gg r'$. Translational symmetry also allows us to represent the Green tensor in wave vector space along z and z' according to

$$\mathcal{G}(\mathbf{r},\mathbf{r}',\omega)=\int\frac{dq}{2\pi}\,\mathcal{G}_{\rm 2D}(\mathbf{R},\mathbf{R}',q,\omega)\,\mathrm{e}^{\mathrm{i}q(z-z')},$$

where the two-dimensional Green tensor $\mathcal{G}_{\rm 2D}(\mathbf{R},\mathbf{R}',q,\omega)$ has the far-field behavior

$$\mathcal{G}_{\rm 2D}(\mathbf{R},\mathbf{R}',q,\omega)\xrightarrow[k_{\rm h}R\gg1]{}\frac{\mathrm{e}^{\mathrm{i}Q_{\rm h}R}}{\sqrt{Q_{\rm h}R}}\mathbf{S}(\hat{\mathbf{R}},\mathbf{R}',q,\omega)$$

with $Q_{\rm h}=\sqrt{k_{\rm h}^2-q^2+\mathrm{i}0^+}$ defined in the same way as in Sec. 4.1 of the main text. Combining the above expressions, we have

$$\mathcal{G}(\mathbf{r},\mathbf{r}',\omega)\xrightarrow[k_{\rm h}R\gg1]{}\int\frac{dq}{2\pi}\frac{\mathrm{e}^{\mathrm{i}(Q_{\rm h}R+qz)}}{\sqrt{Q_{\rm h}R}}\mathrm{e}^{-\mathrm{i}qz'}\mathbf{S}(\hat{\mathbf{R}},\mathbf{R}',q,\omega).$$

We now work out the q integral in the asymptotic limit by following the stationary-phase method. More precisely, we approximate $Q_{\rm h}R+qz\approx k_{\rm h}r-(k_{\rm h}^2R/2Q_0^3)(q-q_0)^2$ by its second-order Taylor expansion around the stationary point defined by the vanishing of its first derivative $-q_0R/Q_0+z=0$ with $Q_0=\sqrt{k_{\rm h}^2-q_0^2}$ (i.e., with $q_0<k_{\rm h}$ such that $(Q_0,q_0)\parallel(R,z)$, and therefore, $Q_0=k_{\rm h}R/r$ and $q_0=k_{\rm h}z/r$). Since only the region very close to q_0 contributes to the integral in the far-field limit, we can set $q=q_0$ in the rest of the integrand and write

$$\mathcal{G}(\mathbf{r},\mathbf{r}',\omega)\xrightarrow[k_{\rm h}R\gg1]{}\frac{\mathrm{e}^{\mathrm{i}k_{\rm h}r}}{2\pi\sqrt{k_{\rm h}R^2/r}}\mathrm{e}^{-\mathrm{i}k_{\rm h}zz'/r}\mathbf{S}(\hat{\mathbf{R}},\mathbf{R}',q_0,\omega)\int dq\,\mathrm{e}^{-\mathrm{i}q^2r^3/2k_{\rm h}R^2}=\frac{\mathrm{e}^{\mathrm{i}k_{\rm h}r}}{r}\mathrm{e}^{-\mathrm{i}k_{\rm h}zz'/r}\frac{\mathrm{e}^{-\mathrm{i}\pi/4}}{\sqrt{2\pi}}\mathbf{S}(\hat{\mathbf{R}},\mathbf{R}',q_0,\omega),\tag{S3}$$

where the right-most expression is obtained by applying the integral $\int_{-\infty}^{\infty}d\theta\,\mathrm{e}^{\mathrm{i}\theta^2}=\sqrt{\pi}\,\mathrm{e}^{-\mathrm{i}\pi/4}$. Comparing Eqs. (S2) and (S3), we find

$$\mathbf{g}(\hat{\mathbf{r}},\mathbf{R}',\omega)=\frac{\mathrm{e}^{-\mathrm{i}\pi/4}}{\sqrt{2\pi}}\mathbf{S}(\hat{\mathbf{R}},\mathbf{R}',q_0,\omega),\tag{S4}$$

where $q_0 = k_h z/r$. We use this relation in the main text to find an explicit expression for $\mathbf{g}(\hat{\mathbf{r}}, \mathbf{R}', \omega)$ based on the far-field limit of the outgoing cylindrical waves generated by a line dipole placed inside the waveguide for the particular case of $q = 0$ (normal emission) with $\mathbf{S}(\hat{\mathbf{R}}, \mathbf{R}', \omega) \equiv \mathbf{S}(\hat{\mathbf{R}}, \mathbf{R}', q = 0, \omega)$. Gathering the results in Secs. 4.2 and 4.3, together with Eq. (S4), we obtain

$$\mathbf{g}(\hat{\mathbf{r}}, \mathbf{R}', \omega) = k^2 \sum_m \mathrm{i}^{-m} J_m(k_1 R')\, \mathrm{e}^{\mathrm{i}m(\varphi-\varphi')} \left[t_{m,pp}\, \hat{\mathbf{z}} \otimes \hat{\mathbf{z}} + \frac{1}{2} \sum_{\pm} t_{m\pm1,ss}\, \hat{\varphi} \otimes (\hat{\varphi} \pm \mathrm{i}\hat{\mathbf{R}}) \right],$$

whose components in the $\{\hat{\mathbf{R}}, \hat{\varphi}, \hat{\mathbf{z}}\}$ frame depend on φ and φ' only through the difference $\varphi - \varphi'$, thus reflecting the cylindrical symmetry of the system.

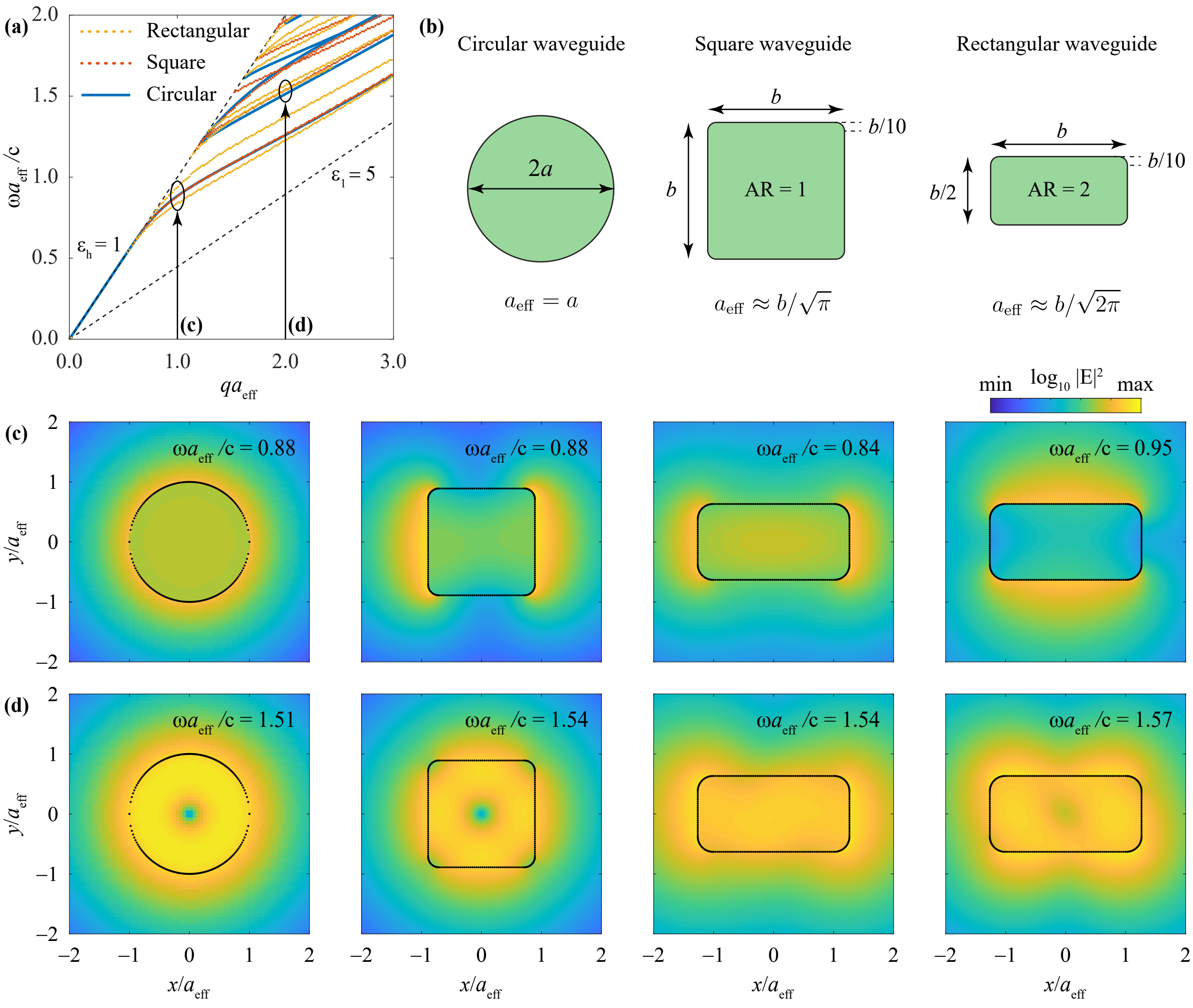

FIG. S2: **Comparison of circular, square, and rectangular waveguide geometries. (a)** Normalized dispersion relation of guided modes in circular (solid-blue curves), square (red-dotted curves), and rectangular (yellow-dotted curves) waveguides, which are parametrized according to the schemes in panel **(b)**, so that their cross-sectional areas are all fixed to a common effective value πa_{eff}^2. In panel (a), only the lowest-order modes that correspond to those presented in Figure S1(a) for the circular waveguide are presented, while the arrows labeled by (c) and (d) indicate the wave vectors at which the intensity profiles (see logarithmic color scale) of the lowest-order modes are investigated in the corresponding panels below for specific values of $qa_{\mathrm{eff}} = 1$ **(c)** and $qa_{\mathrm{eff}} = 2$ **(d)**, with the normalized frequency indicated in the top-right corner of each panel.

S5. RECTANGULAR WAVEGUIDES

While the cylindrical waveguide geometry offers a natural symmetry that facilitates an analytical description of waveguide modes, and thus also their free-space excitation and nonlinear interaction, the proposed down-conversion scheme applies generally to other waveguide geometries. Rectangular waveguides in particular are among the most widely explored morphologies in the context of SPDC, and constitute a more realistic choice for materials with a large anisotropic $\chi^{(2)}$ nonlinearity such as lithium niobate [3, 4]. In Figure S2(a), we compare the dispersion relations of rectangular (dashed-yellow curves) and square (dotted-red curves) waveguides with that of the cylindrical waveguide (solid-blue curves), also presented in Figure S1(a). The specific geometries considered in each case are shown schematically in Figure S2(b), where the radius a and the lateral size b characterize the circular and rectangular geometries, respectively, while an effective radius $a_{\rm eff}$ is introduced in such a way that the cross-section area $\pi a_{\rm eff}^2$ is the same in all of them. We extract the normalized dispersion relation of rectangular and square waveguides from the local density of optical states calculated by using the boundary-element method (BEM) [5], which is found to be only weakly dependent on the specific edge rounding considered. The lowest-order mode supported by the square waveguide emerges in the same energy range as the associated mode in the cylindrical waveguide (cf. the overlapping dotted-red and solid-blue curves), while a rectangular waveguide with an aspect ratio AR $= 2$ lifts the twofold degeneracy of the same mode in the confinement plane. The qualitative similarities exhibited in the waveguide dispersion relations are reflected in the intensity profiles presented in Figure S2(c) at the guided mode frequency indicated in each panel for a selected wave vector q such that $qa_{\rm eff} = 1$. Peaks in the circular and square geometries occur at the approximately same frequency $\omega a_{\rm eff}/c \approx 0.88$, while the rectangular waveguide supports resonances above ($\omega a_{\rm eff}/c = 0.84$) and below ($\omega a_{\rm eff}/c = 0.95$), corresponding to horizontal and vertical cross-sectional confinement, respectively. In Figure S2(d), we plot the intensity profile associated with the second lowest order modes at $qa_{\rm eff} = 2$, which are all characterized by a minimum in the center of the waveguide profile. In this particular instance, the circular mode displays a lower frequency compared to the other shapes. The similarity of the dispersion relation and mode profiles exhibited by these selected geometries indicates that the SPDC scheme introduced in the main text for cylindrical waveguides can be generally applied to other waveguide morphologies.

[1] F. J. García de Abajo, A. Rivacoba, N. Zabala, and P. M. Echenique, Phys. Rev. B **68**, 205105 (2003).

[2] DLMF, *NIST Digital Library of Mathematical Functions*, http://dlmf.nist.gov/, Release 1.1.3 of 2021-09-15, f. W. J. Olver, A. B. Olde Daalhuis, D. W. Lozier, B. I. Schneider, R. F. Boisvert, C. W. Clark, B. R. Miller, B. V. Saunders, H. S. Cohl, and M. A. McClain, eds., URL `http://dlmf.nist.gov/`.

[3] H. Jin, F. Liu, P. Xu, J. Xia, M. Zhong, Y. Yuan, J. Zhou, Y. Gong, W. Wang, and S. Zhu, Phys. Rev. Lett. **113**, 103601 (2014).

[4] Y. Kong, F. Bo, W. Wang, D. Zheng, H. Liu, G. Zhang, R. Rupp, and J. Xu, Adv. Mater. **32**, 1806452 (2020).

[5] F. J. García de Abajo and A. Howie, Phys. Rev. B **65**, 115418 (2002).
